# Impulsivity is Associated with Early Sensory Inhibition in Neurophysiological Processing of Affective Sounds

**DOI:** 10.3389/fpsyt.2015.00141

**Published:** 2015-10-07

**Authors:** Takahiro Soshi, Takamasa Noda, Kumiko Ando, Kanako Nakazawa, Hideki Tsumura, Takayuki Okada

**Affiliations:** ^1^Department of Forensic Psychiatry, National Institute of Mental Health, National Center of Neurology and Psychiatry, Kodaira, Japan; ^2^Department of Psychiatry, National Center Hospital, National Center of Neurology and Psychiatry, Kodaira, Japan

**Keywords:** impulsivity, sensory gating, paired-click paradigm, white noise, auditory-evoked potential, event-related desynchronization/synchronization

## Abstract

Impulsivity is widely related to socially problematic behaviors and psychiatric illness. Previous studies have investigated the relationship between response inhibition and impulsivity. However, no study has intensively examined how impulsivity correlates with automatic sensory processing before the drive for response inhibition to sensory inputs. Sensory gating (SG) is an automatic inhibitory function that attenuates the neural response to redundant sensory information and protects higher cognitive functions from the burst of information processing. Although SG functions abnormally in several clinical populations, there is very little evidence supporting SG changes in conjunction with impulsivity traits in non-clinical populations. The present study recruited healthy adults (*n* = 23) to conduct a neurophysiological experiment using a paired-click paradigm and self-report scales assessing impulsive behavioral traits. Auditory stimuli included not only a pure tone but also white noise to explore the differences in auditory-evoked potential (AEP) responses between the two stimuli. White noise is more affective than pure tones; therefore, we predicted that the SG of AEPs (P50, N100, and P200) for white noise would correlate more with self-reported impulsivity than with those for pure tones. Our main findings showed that SG of the P50 and P200 amplitudes significantly correlated with self-reported reward responsiveness and fun-seeking, respectively, only for white noise stimuli, demonstrating that higher-scoring impulsivity subcomponents were related to greater SG. Frequency-domain analyses also revealed that greater desynchronization of the beta band for the second white noise stimulus was associated with higher motor impulsivity scores, suggesting that an impulsivity-related change of SG was associated with attentional modulation. These findings indicate that the measurement of SG of white noise may be an efficient tool to evaluate impulsivity in non-clinical populations, and should also be applied to clinical populations.

## Introduction

Impulsivity is a complex behavioral trait and has been neurobehaviorally conceptualized as “a predisposition toward rapid, unplanned reactions to internal or external stimuli without regard to the negative consequences of these reactions to the impulsive individual or to others” (1). Impulsivity is related to various behavioral patterns, such as lack of concentration, disinhibition, lack of future planning, sensation seeking, and risk taking (2). Within the clinical domain, impulsivity lies behind antisocial behaviors, such as violence, substance abuse, and suicide (1). Impulsivity is also associated with various psychiatric disorders, including antisocial personality disorder (ASPD) (3), attention-deficit hyperactivity disorder (ADHD) (4), borderline personality disorder (5), and substance addiction (6). Within forensic psychiatry, impulsivity is often associated with aggressiveness; impulsive aggressiveness in patients may lead to problematic behaviors (7). These observations suggest the need for efficient methods to objectively evaluate impulsivity in various populations.

Neural correlates of impulsivity have often been explored using a response inhibition task related to higher cognitive control (5, 8–10). However, impulsivity also underlies a pre-attentive, rash response (11), and may affect automatic sensory processing before response inhibition takes place. Several personality models have been proposed (3, 12–14) that generally argue that impulsivity is related to early sensory processing. That is, people with low impulsivity tend to possess high sensitivity to warning signals and are tolerant to the attenuation of simple behavioral performance (12, 15). Taken together, impulsivity may affect automatic sensory processing of warning signals, such as affective stimuli.

One of the more suitable experimental paradigms for the investigation of automatic sensory processing is sensory gating (SG) (16, 17). SG filters out redundant information and extracts prominent sensory information from the environment. To evaluate auditory SG, a paired-click paradigm has been frequently utilized (17–19): the first conditioning (S1) and second testing (S2) sounds with various durations (0.04–40 ms) (16, 20) successively stimulate participants with various stimulus intervals (e.g., 75, 150, and 500 ms) (18). Cortical inhibitory mechanisms attenuate neuronal activation to the S2, compared with the S1. SG is mainly observed in the auditory-evoked potential (AEP) waveform labeled P50, which is a positive-voltage response peaking around 50 ms after stimulus onset. SG of P50 is associated with various cortical areas (21). An intracranial electroencephalogram (EEG) study has observed SG in the inferior temporo-parietal areas, supplementary motor area, and the adjacent anterior cingulate cortex, and hippocampus (22, 23). Another intracranial study estimated that the SG of P50 was related to the dorsolateral prefrontal areas (24). Additionally, a neuroimaging study reported that SG was related to several areas including the prefrontal, superior temporal, parietal, thalamus, insula, and hippocampus (25, 26).

Clinical studies have reported that SG functions abnormally in patients with current and developing psychiatric illness (16, 27). Patients with schizophrenia, for example, showed higher S2–S1 ratios, that is, lower SG, of the P50 component than non-schizophrenic controls (17, 22, 28–30), and attenuated activations in the bilateral thalamus and hippocampus (31). The SG deficit is likely related to malfunctions of early sensory and attentional processing, which may be related to modulation in the α7 nicotinic receptor system (32). Micoulaud-Franchi et al. (28) also compared SGs among patients with schizophrenia, ADHD, or healthy controls. They observed that patients with schizophrenia or ADHD showed attenuated SGs compared with the healthy controls; furthermore, patients with ADHD showed lower SG than those with schizophrenia. Another study reported that SG of P50 in disgust contexts significantly correlated with social functioning scores in patients with bipolar disorder (33). To summarize, SG deficits may be associated with both defective automatic and attentive sensory processing (34), and SG in affective contexts may sensitively correlate with behavioral assessments in clinical populations.

Despite accumulated findings concerning SG in specific clinical populations, the relationship between SG and impulsivity has not been investigated intensively, with the exception of studies by one research group (3, 11, 35). Lijffijt et al. (3), for example, recruited impulsive patients with ASPD and healthy controls and conducted a paired-click experiment with pure tone stimuli. They did not observe a difference in the mean SG ratios (S2/S1) between the two groups, but reported group differences in correlations between SG ratios and self-reported impulsivity. Healthy controls showed a negative correlation between P50 ratios and impulsivity scores, while patients with ASPD showed a positive correlation between P50 ratios and impulsivity scores. This finding suggests that impulsivity is related to SG, and differently affects SG in populations with different impulsivity traits.

The present study involved an EEG experiment using a paired-click paradigm. Healthy participants were stimulated by two types of randomized auditory stimulus pairs: pure tones and white noise. Studies have shown that white noise is less pleasant than pure tones (36) and may, therefore, function as a warning ­affective signal. Hence, the two sound types may differentially affect SG of P50 and other AEPs (N100 and P200) (37, 38). Because SG tends to be attenuated under attention, and reduced impulsivity may be related to increased attentive affective processing (39, 40) and increase sensitivity to warning signals (12, 15), SG may be attenuated in people with fewer or reduced impulsivity scores. This may be most apparent under the white noise condition. Additionally, we conducted frequency-domain analyses to evaluate power changes in the major frequency bands: delta (δ), theta (θ), alpha (α), beta (β), and gamma (γ). Based on previous studies, attention is related to α (41) or β (42) bands, therefore impulsivity scores may significantly correlate with power changes in these frequency bands for the white noise condition more so than for the pure tone condition.

## Materials and Methods

### Participants

Twenty-three healthy Japanese adults (16 women and 7 men) participated in the experiment. The mean age and educational years of the men (age: mean ± SD, 26.1 ± 8.5 years old; education: 17.1 ± 4.1 years) and women (30.7 ± 10.4 years old; 15.9 ± 2.4 years) were similar (Mann–Whitney: age, *U* = 40.0, *p* = 0.308; education: *U* = 53.0, *p* = 0.871; Table 1). Their psychiatric histories were assessed according to the SCID-I/NP (Structured Clinical Interview for DSM-IV-TR Axis I Disorders, Non-patient Edition) (43) by a psychiatrist or clinical psychologist. None of the participants violated the exclusion criteria, including history of psychiatric illness, brain damage, cognitive deficits, substance abuse, or inability to understand Japanese. In addition, they did not habitually smoke cigarettes, which could affect SG of the P50 component (44, 45). All participants confirmed their own normal hearing abilities at a periodical health examination and had normal or corrected-to-normal vision. Right-handedness was assessed using the Edinburgh handedness inventory (46). The present study was conducted according to the guidelines of the Helsinki Declaration. Participants provided written informed consent based on the protocol approved by the Ethical Committee of the National Center of Neurology and Psychiatry (NCNP).

**Table 1:** **Demographic and self-reported impulsivity profiles of healthy participants (*n* = 23)**.

	Women (*n* = 16)		Men (*n* = 7)	
	
	Mean	SD	Mean	SD
Age (years)	26.1	8.5	30.7	10.4
Education (years)	17.1	4.1	15.9	2.4
**BIS-11**				
AI	15.3	3.7	14.3	2.7
MI	23.9	4.7	20.5	2.2
NPI	26.7	1.9	25.1	3.5
**BIS/BAS**				
BIS	18.1	5.1	21.2	2.9
D	12.4	1.5	11.7	1.6
RR	17.4	2.1	16.1	2.5
FS	12.6	2.0	10.2	1.9

*AI, attentional impulsivity; MI, motor impulsivity; NPI, non-planning impulsivity; BIS/BAS, behavioral inhibition/activation system; D, drive; RR, reward responsiveness; FS, fun-seeking*.

### Paired-click paradigm and auditory stimuli

**Figure 1:**
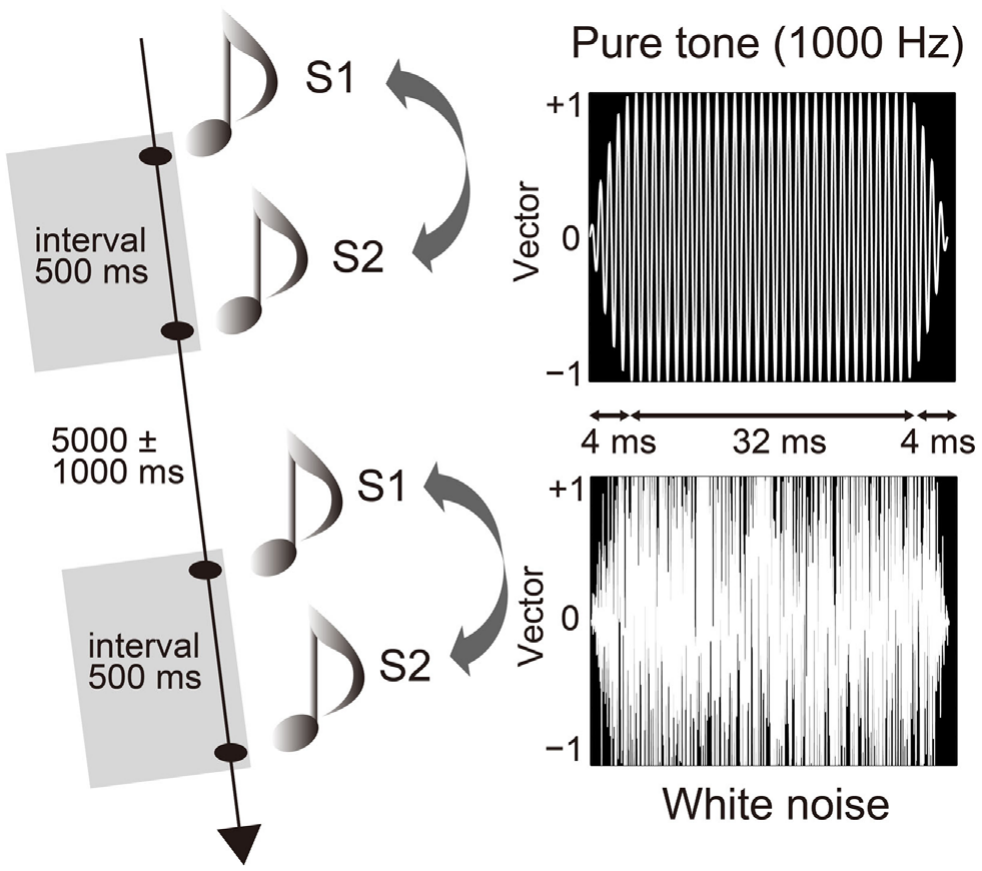
**Paired-click paradigm and auditory stimuli**. The first (S1) and second (S2) stimuli successively stimulated participants with an interval of 500 ms and an inter-trial interval of 5000 ± 1000 ms. Stimulus pairs of pure tone (1000 Hz) and white noise were presented randomly and in equal frequency. Duration of each stimulus was 40 ms with a plateau of 32 ms and a rise and fall of 4 ms.

Participants sat on a comfortable chair inside a sound attenuated chamber [about 38 dB sound pressure level (SPL)]. They performed a paired-click paradigm, in which the S1 and S2 sounds were successively presented with a fixed interval of 500 ms (20) and an inter-trial interval of 5000 ± 1000 ms (Figure 1). Each of the three trial blocks consisted of 60 pairs (a total of 180 pairs). Participants were instructed to passively listen to sounds through headphones (DR-531, Elega Acous Co. Ltd., Tokyo, Japan) (47) with their eyes closed (48). The auditory stimuli consisted of either a pair of pure tones (1000 Hz) or a pair of white noise tones (Figure 1). While the click stimuli (duration of 4 ms) produced from the white noise were also used in previous studies (49), we used a longer duration of white noise, and then compared it with a pure tone. Each sound stimulus was presented randomly and equally as often in each block (MTS0410, Medical Try System, Tokyo, Japan) for 40 ms, with a plateau of 32 ms and a rise/fall of 4 ms (20). Auditory stimuli were calibrated to about 72 dB SPL around the ear canal entrance. The pure tone was sampled monaurally at 44,100 Hz, 16-bit. White Gaussian noise was sampled with the same sampling parameters. The rise and fall of the sounds were produced using a raised-cosine filter. After the experiment, participants answered according to a 10-point hedonic Likert scale concerning the stimuli (10 = “very pleasant”; 1 = “very unpleasant”). It was confirmed that white noise was more unpleasant than the pure tone (pure tone: 7.9 ± 1.3; white noise: 4.3 ± 2.5; Wilcoxon, *Z* = 2.808, *p* = 0.005).

### Impulsivity measurement

Impulsivity traits were measured with the Barratt Impulsiveness Scale (BIS-11) (50, 51). The BIS-11 contains 30 items, and is scored using a four-point Likert scale (4 = “very true for me”; 3 = “somewhat true for me”; 2 = “somewhat false for me”; and 1 = “very false for me”). Three subcomponents were utilized: attentional impulsivity (AI: 8–32 scores), motor impulsivity (MI: 11–44 scores), and non-planning impulsivity (NPI: 10–40 scores). AI is characterized as lack of concentration. MI is concerned with actions without consideration. The NPI is defined as a lack of planning for the future (51).

Behavioral inhibition properties associated with impulsivity were also scored by the behavioral inhibition/activation systems (BIS/BAS) scales (52, 53). The BIS/BAS included 20 items, and was answered similarly using a four-point Likert scale. The BIS component (7–28 scores) is associated with avoidance of negative behavioral results. The BAS assesses motivational preference for pleasant consequences: drive (D: 4–16 scores), reward responsiveness (RR: 5–20 scores), and fun-seeking (FS: 4–16 scores).

In both scales, greater scores indicate more salient self-reported behavioral traits. Each score of the female and male participants is summarized in Table 1.

### Electroencephalogram recording and analyses

#### Data Recording

Electroencephalograms (1000 ms pre-stimulus to 1000 ms post-stimulus) were recorded from the four midline scalp Ag/AgCl electrodes (Fz, Cz, Pz, and Oz) with a commercialized EEG recording system (MEB-2300; NIHON KODEN Corp., Tokyo, Japan). Three electrodes were also placed at the outer canthi of the eyes for monitoring horizontal (HEOG: left-upper minus right-upper) and vertical (VEOG: left-upper minus left-lower) electro-oculograms. All electrodes were referenced to the linked mastoids. The ground electrode was positioned on the participants’ chin. EEGs were recorded at a sampling frequency of 1024 Hz, and were amplified with band-pass filter of 0.1–100 Hz. The impedance was set below 5 kΩ throughout the experiment.

#### Time-Domain Analysis

Raw EEGs were filtered offline with a band-pass filter of 0.5–40 Hz. VEOGs were subtracted from the individual epochs by a regression method (54). Regression coefficients (*β*) were firstly calculated for individual VEOGs (mEEG = *β* × VEOG + *C*; mEEG: measured EEG; *C*: intercept of the equation). Subsequently, VEOGs were subtracted from the EEGs (estEEG = mEEG − *β* × VEOG; estEEG: estimated EEG). After reduction of the VEOG artifacts, 90 epochs were obtained for both S1 and S2 in the white noise and pure tone conditions, from 100 ms before stimulus onset to 400 ms after stimulus onset. Individual averaged waveforms were calculated after baseline correction (mean potentials during the baseline interval from −100 to 0 ms) and artifact rejection for residual artifacts (±75 μV). Mean rejection rates were about 3%. Individual averaged waveforms were high-pass filtered at 10 Hz again to specifically examine P50 gating (16, 37, 55).

SG of the AEPs was calculated with the equation: S2/S1 × 100. Proportional scores below 100 indicate greater SG. AEPs were specified for individual participants at the Cz position (56). The first positive AEP, P30, was defined as the positivity peak during the interval from about 20 to 40 ms post-stimulus and was used as a landmark to specify subsequent components (16, 57). The following positive AEP was the P50, defined as a peak in the time window from about 40 to 80 ms (18). The next negative component, the N100, was identified as the negative peak in the time window from 80 to 150 ms. The following positive component, the P200, was defined as the positive peak in the time window from 150 to 250 ms. Peak amplitudes of P50, N100, and P200 were defined as the voltage values from the preceding trough to the component peak (58–60). When preceding troughs were not clearly observed, peak amplitudes were calculated from the adjacent minimal slope (58), or the nearest trough.

#### Frequency-Domain Analysis

We also performed a frequency-domain analysis to examine event-related synchronization/desynchronization (ERS/ERD), which reflects an increase or decrease, respectively, in the synchrony of neuronal activities time-locked to stimulus events (61). ERS/ERD is represented by relative powers of event-related neural activities to reference periods before the stimulus onset: ERS/ERD (%) = (*A* – *R*)/*R* × 100 (*A* = band powers of event-related activities; *R* = band powers of reference periods). Unlike the time-domain analysis, raw EEGs (0.1–100 Hz) were not offline-filtered. VEOG reduction was conducted for each individual EEG, and EEG epochs were spliced out for reference frame (−1000 to the S1 onset), S1 (the onset to 300 ms), and S2 (the onset to 300 ms) for both the pure tone and white noise. Duration of the common reference period was determined by the data acquisition setting of the recording system (−1000 ms before S1 onset), and the counterparts of S1 and S2 were determined to comprise P50, N100, and P200 components. Each epoch was converted into amplitude (real number part) and phase (complex number part) by Fourier transformation. To equate a base frequency (0.977 Hz), S1 and S2 epochs were transformed with the resolution of 1024 data points by attaching 0s behind recorded data. The power (dB) of each frequency domain was calculated by the equation: power (dB) = 20 × log_10_ [Abs (fft)] [Abs (fft) = a real number part of each frequency domain (fft)], and was averaged in a reference frame, S1, and S2. ERS/ERD scores were finally obtained for S1 and S2 by the equation mentioned above.

#### Statistical Analysis

For the time-domain data, S1 and S2 amplitudes were compared by a two-way repeated-measures analysis of variance (ANOVA) with stimulus type (ST) (pure tone and white noise) and order (S1 and S2) as factors for P50, N100, and P200. When significant interaction effects were found, follow-up ANOVAs were performed to examine the order effect for each ST. The P50 to S2 and P200 to S1 in the pure tone condition, and the P200 to S2 in the white noise condition violated normality (Kolmogorov–Smirnov test); therefore, all amplitude data were transformed into natural logarithms to satisfy normality (*ps* > 0.131).

S2–S1 ratios for the pure tone and white noise conditions were compared with paired *t*-tests separately for P50, N100, and P200. None of the S2–S1 ratios violated normality (*ps* > 0.147) and they were not transformed logarithmically.

The relationships between S2–S1 ratios and impulsivity scores were examined by correlation analyses with a permutation procedure (62) to avoid type I errors in multiple analyses (6 SG scores × 7 impulsivity scores). This non-parametric permutation procedure relies on avoiding overestimation by testing distributions of statistical values that are empirically obtained by multiple permutation analyses from collected samples. S2–S1 ratios and impulsivity scores were randomly and repeatedly paired across the 23 participants and were correlated by Pearson’s method, and a total of 10000 coefficients (*r*) were obtained. We tested whether actual coefficients were outside the 95% confidence intervals (CIs) of the permutation distributions of the coefficients (*p* < 0.05). Significant correlations were also tested using permutation procedures under the control of age and sex, because these factors may affect SG (56, 63). MI and RR scores violated normality (*ps* < 0.025); therefore, all behavioral trait scores were transformed logarithmically.

For the frequency-domain data, ERS/ERD values of major frequency bands were first calculated for S1 and S2 (δ: 1–4 Hz; θ: 4–8 Hz; α: 8–12 Hz; β: 12–30 Hz; and γ: >30 Hz) (64). The upper frequency of γ band was set to 50 Hz (the commercial frequency in Eastern Japan) to avoid contamination of line noise. ERS/ERD values of S1 and S2 were compared by two-way ANOVAs with within-participant factors of ST (pure tone and white noise) and order (S1 and S2) for each frequency domain. When significant interaction effects appeared, follow-up ANOVAs examined a main effect of order for each ST. ERS/ERD values satisfied normality (*ps* > 0.098), and hence, raw values were used for ANOVAs.

We also performed permutation correlation analyses to examine the relationships between ERS/ERD effects for S2 and impulsivity scores. ERS/ERD effects for S2 were defined as the difference between S2 and S1 (S2 − S1). We did not use proportional scores (S2/S1 × 100), because ERS/ERD may potentially be represented by both positive and negative values. More negative difference scores represent larger desynchronization of frequency bands for S2, as compared to S1. Difference ERS/ERD values and impulsivity scores were randomly correlated across the participants, and 10000 dummy coefficients were obtained. Actual coefficients outside 95% CIs were considered significant (*p* < 0.05). Significant correlations were also tested using the covariates of age and sex. All impulsivity scores were transformed logarithmically.

## Results

### Time-domain analysis

The averaged scores of the three subcomponents of the BIS-11 were 14.6 ± 3.0 for AI, 21.5 ± 3.4 for MI, and 25.6 ± 3.1 for NPI. Scores of the BIS/BAS were 20.3 ± 3.9 for BIS, 11.9 ± 1.6 for D, 16.5 ± 2.5 for RR, and 10.9 ± 2.2 for FS. The S2–S1 ratios from the pure tone and the white noise conditions are summarized in Table 2.

**Table 2:** **Mean amplitude (microvolts) and sensory gating (SG) scores (S2–S1 ratio) of auditory-evoked potentials (*n* = 23)**.

Stimuli		P50		N100		P200	
		Microvolts	SG	Microvolts	SG	Microvolts	SG
Pure tone	S1	1.72	64	−5.00	60	7.27	57
	S2	1.10		−2.97		4.15	
White noise	S1	2.11	45	−4.39	47	6.65	55
	S2	0.95		−2.08		3.65	

*S1, the first stimulus; S2, the second stimulus*.

**Figure 2:**
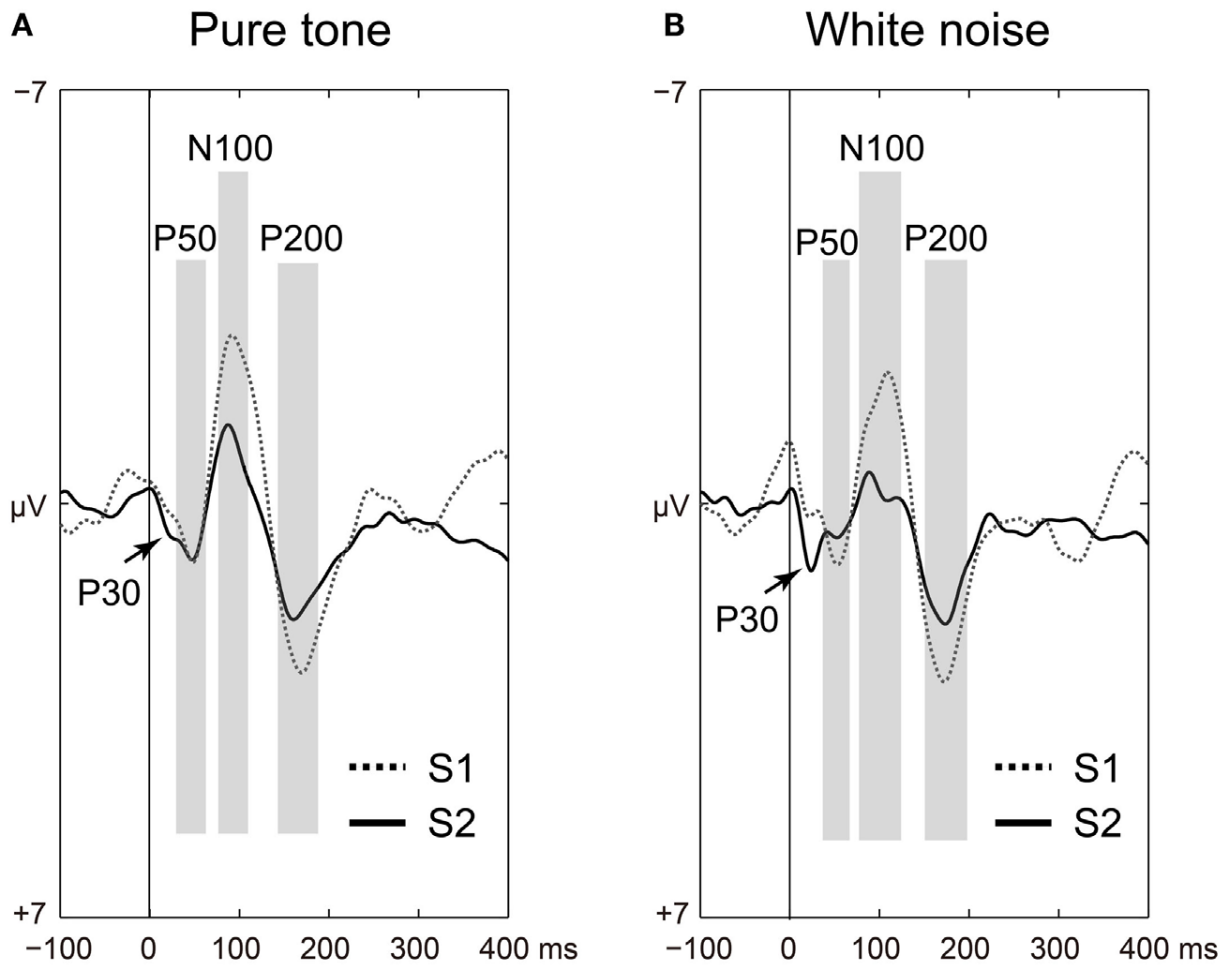
**Time-domain analyses of the first (S1: broken line) and second (S2: solid line) stimuli compared for (A) pure tones and (B) white noise**. Auditory-evoked potential components (P50, N100, and P200) are shaded in light gray. Irrespective of stimulus type, sensory gating (reduced S2 amplitudes from preceding troughs to peaks) is significantly observed in all of P50, N100, and P200 (*ps* < 0.0001).

The P50, N100, and P200 amplitudes were compared between the S1 and S2 time points for the pure tones (Figure 2A) and white noise (Figure 2B). The ANOVAs for ST and order (O) demonstrated that SG effects (order effects) were observed in all components of P50 [O: *F*(1,22) = 50.438, *p* < 0.0001; ST × O: *F*(1,22) = 1.873, *p* = 0.185], N100 [O: *F*(1,22) = 72.453, *p* < 0.0001; ST × O: *F*(1,22) = 2.021, *p* = 0.169], and P200 [O: *F*(1,22) = 159.531, *p* < 0.0001; ST × O: *F*(1,22) = 0.395, *p* = 0.536]. The N100 and P200 amplitudes from the pure tone were generally greater than those from the white noise [N100: ST, *F*(1,22) = 4.649, *p* = 0.042; P200: ST, *F*(1,22) = 4.488, *p* = 0.046]. The S2–S1 ratios were not significantly different between pure tones and white noise [P50: *t*(22) = 1.550, *p* = 0.135; N100: *t*(22) = 2.029, *p* = 0.054; P200: *t*(22) = 0.516, *p* = 0.611].

**Figure 3:**
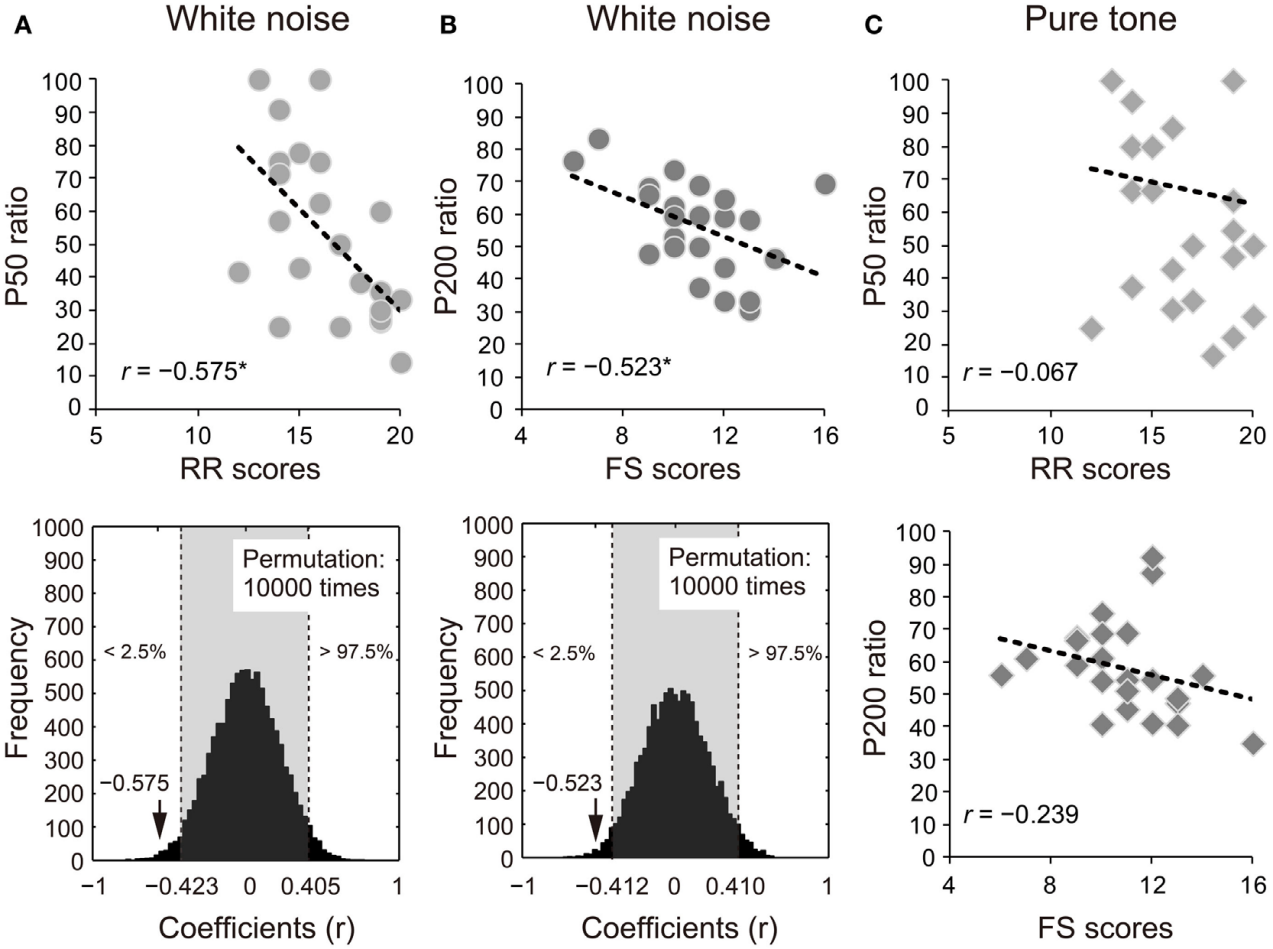
**Proportional scores (stimulus2/stimulus1 × 100) of (A) P50 and (B) P200 for white noise negatively correlate with self-reported reward responsiveness (RR) and fun-seeking (FS), respectively (upper diagrams)**. The actual coefficients (P50: *r* = −0.575; P200: *r* = −0.523) are outside 95% confidence intervals (light gray areas) obtained by permutation correlation analyses (lower diagrams). **(C)** P50 and P200 ratios for pure tones correlated with self-reported RR and FS, respectively for ease of reference.

The ratios of white noise significantly correlated with self-reported RR or FS in P50 (RR: *r* = −0.575, 95% CI: −0.423 to 0.405; *p* < 0.05; Figure 3A) and P200 (FS: *r* = −0.523, 95% CI: −0.412 to 0.410; *p* < 0.05; Figure 3B). The correlations remained significant with the covariates of age and sex (P50: RR, *r*_xy⋅z_ = −0.507, 95% CI: −0.404 to 0.425, *p* < 0.05; P200: FS, *r*_xy⋅z_ = −0.536, 95% CI: −0.425 to 0.425; *p* < 0.05). Conversely, the S2–S1 ratios of pure tones did not show similar correlation properties (P50: RR, *r* = −0.067, 95% CI: −0.461 to 0.395; *p* > 0.05; P200: FS, *r* = −0.239, 95% CI: −0.430 to 0.400; *p* > 0.05; Figure 3C).

### Frequency-domain analysis

**Figure 4:**
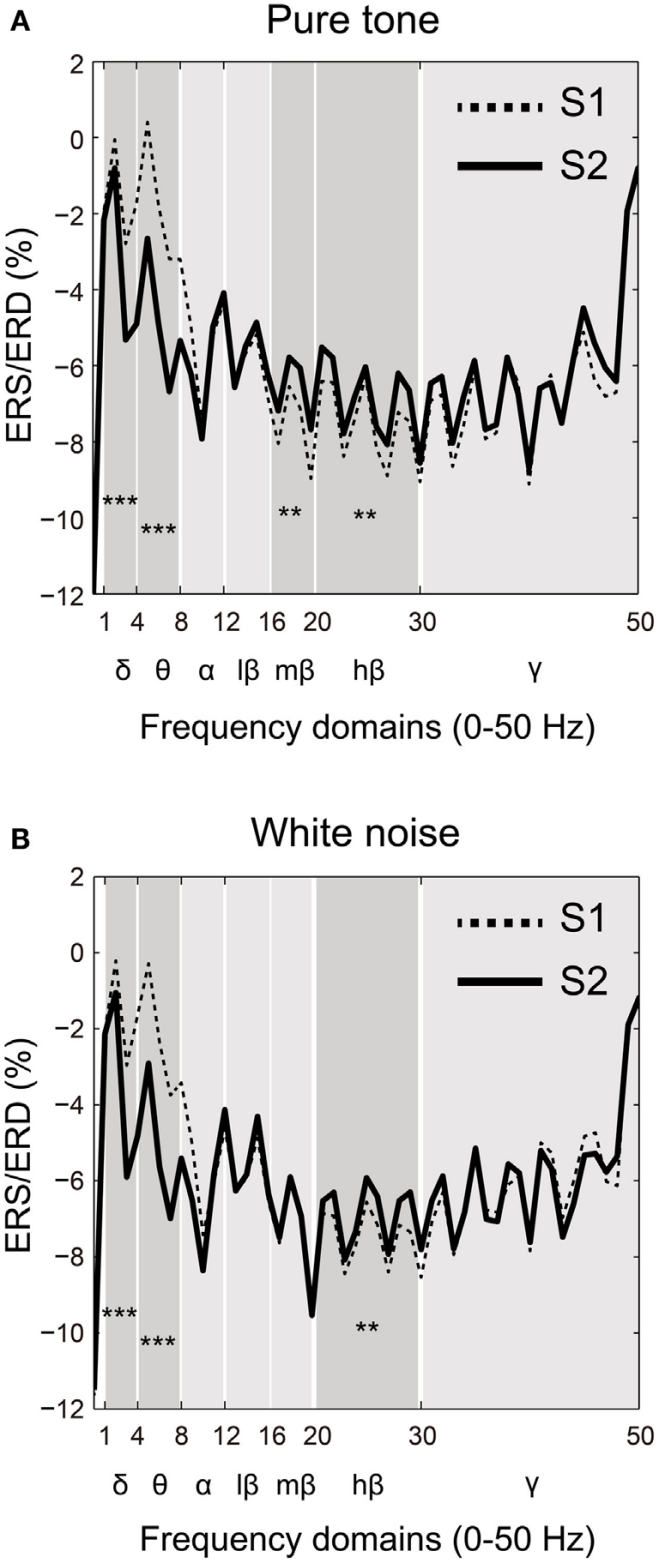
**Frequency-domain analyses of the first (S1: broken line) and second (S2: solid line) stimuli compared for (A) pure tone and (B) white noise**. Power changes of major frequency bands (δ: 1–4 Hz; θ: 4–8 Hz; α: 8–12 Hz; β: 12–30 Hz; and γ: >30 Hz) were represented by event-related synchronization or desynchronization (ERS/ERD). A β band was also separated into low (12–16 Hz), middle (16–20 Hz), and high (20–30 Hz) ranges. For both pure tone and white noise, δ and θ bands for S2 showed greater (more negative) ERD than those for S1, and a high β band for S2 conversely showed smaller (more positive) ERD than for S1. Unlike white noise, pure tone yielded smaller ERD of a middle β band for S2 than for S1.

Event-related synchronization/desynchronization was compared between the S1 and S2 time points for each frequency band (δ, θ, α, β, and γ) in both the pure tone and white noise conditions. Based on a visual inspection of the ERS/ERD waveforms (Figures 4A,B), the lower frequency domains of the β band showed differences in SG between the pure tone and white noise. Accordingly, the β band was subdivided into three frequency bands: low (12–16 Hz), middle (16–20 Hz), and high (20–30 Hz) β bands.

Two-way ANOVAs were conducted for each frequency band with factors of ST (pure tone and white noise) and order (S1 and S2). As can be observed in Figures 4A,B, significant main effects of order were observed for the δ band [O: *F*(1,22) = 71.275, *p* < 0.0001; ST: *F*(1,22) = 0.004, *p* = 0.953; ST × O: *F*(1,22) = 0.009, *p* = 0.927] and for the θ band [O: *F*(1,22) = 122.177, *p* < 0.0001; ST: *F*(1,22) = 1.237, *p* = 0.278; ST × O: *F*(1,22) = 0.188, *p* = 0.669]. Conversely, the high β bands for pure tones and white noise showed smaller (more positive) ERD for S2 than S1 [O: *F*(1,22) = 8.927, *p* = 0.007; ST: *F*(1,22) = 0.015, *p* = 0.904; ST × O: *F*(1,22) = 0.077, *p* = 0.784]. These data demonstrate that the lower frequency bands for S2 showed decreased power compared to S1, and the higher β band reversely showed less attenuation of power for S2, compared to S1.

The middle β band showed differences in ERS/ERD changes between the pure tones and white noise. The two-way ANOVA yielded a significant order effect and a significant interaction in ST × order [O: *F*(1,22) = 4.615, *p* = 0.043; ST: *F*(1,22) = 0.862, *p* = 0.363; ST × O: *F*(1,22) = 6.205, *p* = 0.021]. Follow-up ANOVAs for each ST yielded a significant main effect of stimulus order for the pure tone but not for the white noise [pure tone: *F*(1,22) = 8.369, *p* = 0.008; white noise: *F*(1,22) = 0.030, *p* = 0.863]. These results confirm that S2 in the pure tone condition showed smaller ERD of the middle β band, compared with S1, and that the power of S2 for the white noise condition was not significantly different from that of S1.

The remaining α, low β, and γ bands did not demonstrate any significant effect [α: O, *F*(1,22) = 1.830, *p* = 0.190; ST: *F*(1,22) = 2.662, *p* = 0.117; ST × O: *F*(1,22) = 0.136, *p* = 0.716; low β: O, *F*(1,22) = 1.416, *p* = 0.247; ST: *F*(1,22) = 0.062, *p* = 0.802; ST × O: *F*(1,22) = 0.005, *p* = 0.943; γ: O, *F*(1,22) = 0.687, *p* = 0.416; ST: *F*(1,22) = 2.661, *p* = 0.117; ST × O: *F*(1,22) = 1.951, *p* = 0.176].

**Figure 5:**
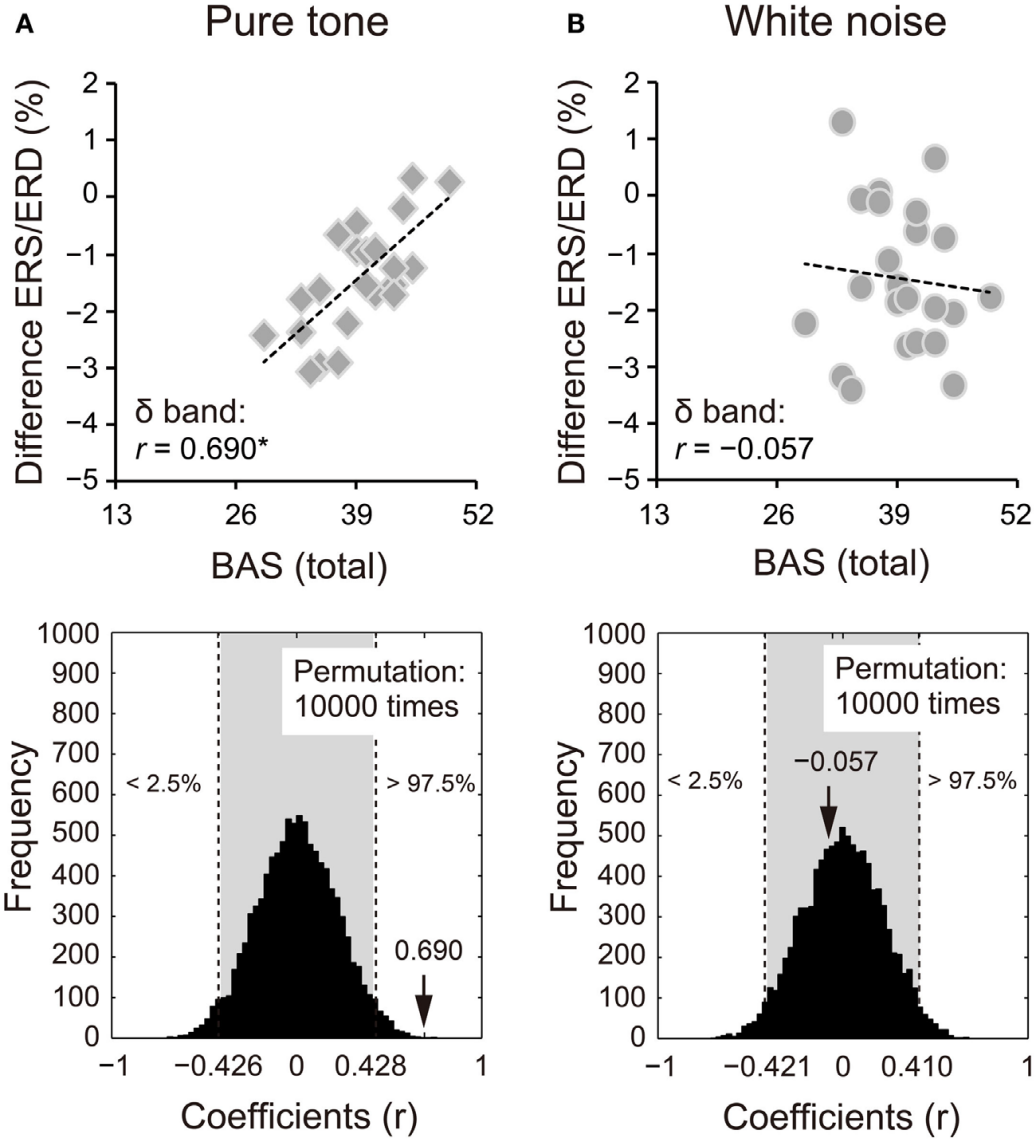
**Power changes in δ bands (1–4 Hz) of the second sounds for (A) pure tone and (B) white noise correlated with total scores of the behavioral activation systems (BAS) components**. Power changes of δ bands were calculated by subtraction of event-related desynchronization (ERD) scores for the first stimuli from ERD scores for the second stimuli. That is, more positive scores indicate smaller ERD in S2, compared with S1. Pure tone showed a positive correlation between power change and total BAS scores, suggesting that higher impulsivity yielded smaller ERD for S2. The actual coefficient (*r* = 0.690) is outside the 95% confidence interval (light gray area) obtained by permutation analyses [lower diagram in **(A)**].

**Figure 6:**
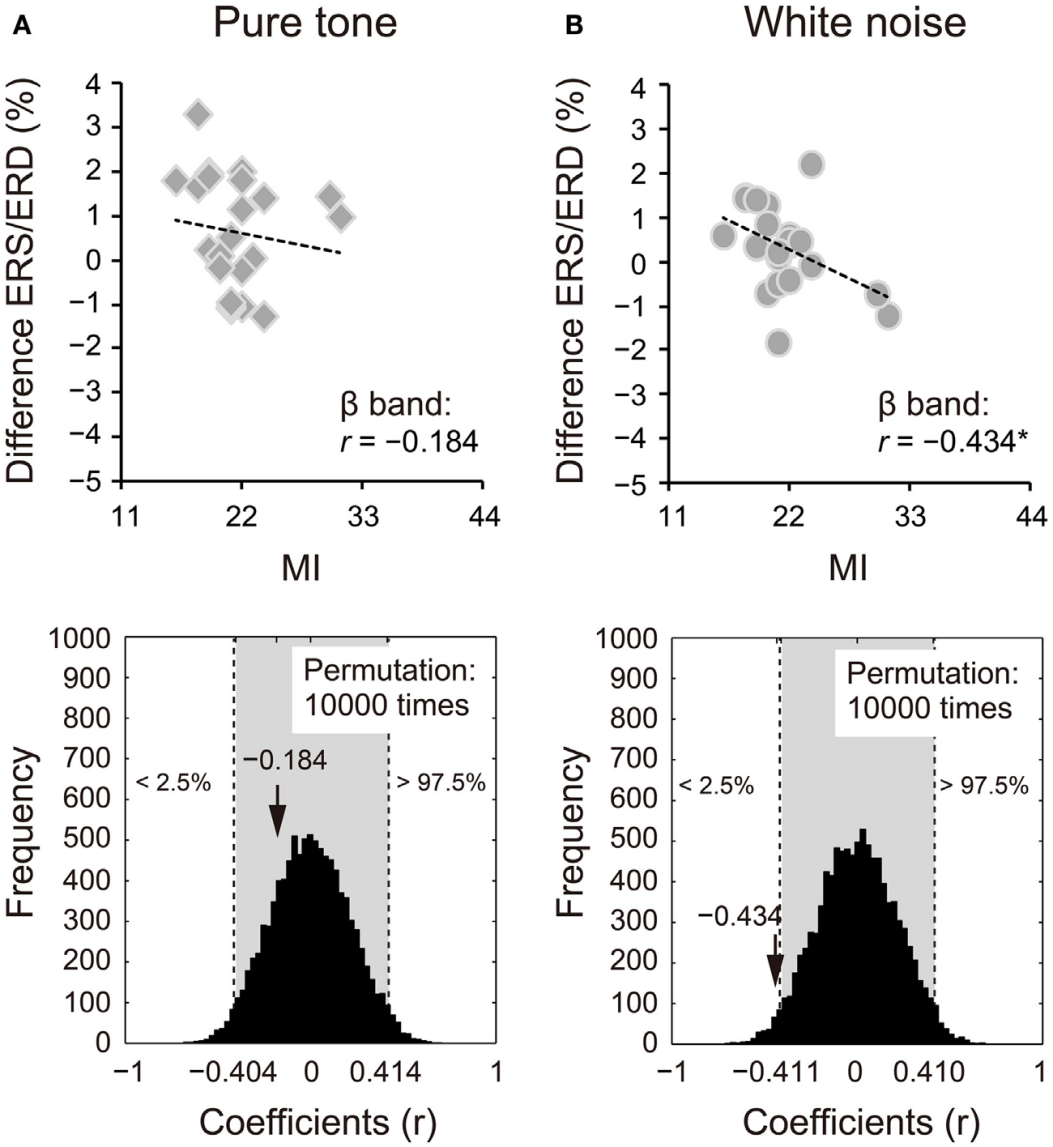
**Power changes in β bands (12–30 Hz) of the second sounds for (A) pure tone and (B) white noise correlated with self-reported motor impulsivity (MI)**. Power changes of β bands were calculated by subtraction of event-related desynchronization (ERD) scores for the first stimuli from those for the second stimuli. White noise showed a negative correlation between power change and self-reported MI, indicating that higher MI scores are associated with greater (more negative) ERD for S2. The actual coefficient (*r* = −0.434) is outside the 95% confident interval (light gray area) of the permutation distribution [lower diagram in **(B)**].

Because we observed differences in the ERS/ERD between S1 and S2 for the δ and β bands, power changes (S2 − S1) of these frequency bands were correlated with impulsivity scores. Power changes in the δ band of S2 for the pure tone positively correlated with the BAS subcomponents (D: *r* = 0.424, 95% CI: −0.405 to 0.408, *p* < 0.05; RR: *r* = 0.570, 95% CI: −0.416 to 0.413, *p* < 0.05; FS: *r* = 0.559, 95% CI: −0.415 to 0.412, *p* < 0.05). A summary of the correlations with the total BAS scores is represented for the pure tone and white noise in Figures 5A,B, respectively (pure tone: *r* = 0.690, 95% CI: −0.402 to 0.408, *p* < 0.05; white noise: *r* = −0.057, 95% CI: −0.422 to 0.409, *p* > 0.05). The summary correlation for the pure tone was also significant when age and sex were controlled (*r*_xy⋅z_ = 0.507, 95% CI: −0.425 to 0.433, *p* < 0.05). For the white noise, power changes in the β band (a total of low, middle, and high β bands) significantly correlated with self-reported MI (*r* = −0.434, 95% CI: −0.411 to 0.410, *p* < 0.05; Figures 6A,B). After elimination of age and sex effects, the correlation tended to be significant (*r*_xy⋅z_ = −0.382, 95% CI: −0.434 to 0.423, *p* < 0.1). These findings suggest that pure tones and white noise were associated with different ERS/ERD changes modulated by impulsivity subcomponents.

## Discussion

Unlike previous studies, our study used both white noise and a pure tone paradigm to examine the differences in SG changes with self-reported impulsivity in non-clinical, healthy persons.

Consistent with previous studies (3), the present study also demonstrated that SG significantly correlates with self-reported impulsivity in healthy adults. To our knowledge, we are the first to observe that SG to white noise but not to a pure tone significantly correlated with impulsivity components. It has been suggested, on the other hand, that a high-pass filter with frequency bands lower than 10 Hz affects SG of P50 (20, 65), and may diminish differences in SG between a pure tone and white noise. However, we observed different correlations with self-reported impulsivity between the pure tone and white noise conditions under the ordinary high-pass filter setting. Additionally, the frequency-domain analyses demonstrated that δ and θ bands activities for S2, compared with those for S1, increased desynchronizations similarly for the pure tone and white noise conditions. These suggest that the present time-domain analyses detected SG differences between the two types of sounds. To summarize, when different types of sounds are presented concurrently, self-reported impulsivity is likely more sensitively associated with SG of more affective sounds in a pre-attentive level (39).

The P50 ratio in the white noise condition negatively correlated with the self-reported affective RR, that is, a greater SG of P50 (smaller S2–S1 ratios) was related to higher self-reported RR. As observed in Figure 3C, however, there are some outlier-like data points in the pure tone condition (RR = 19, SG = 100; RR = 12, SG = 25), which might prevent the significant correlation. To examine the possibility, we conducted additional correlation analyses without the two outlier-like data. The correlation still did not reach significance for pure tones (*r* = −0.204, *p* = 0.376). Conversely, the corresponding analysis excluding the same two persons’ data points yielded a significant correlation for white noise (*r* = −0.719, *p* = 0.0002). These supplementary tests suggest that the pure tone and white noise conditions yielded different impulsivity-related SG modulations.

Enhancement of P50 SG in healthy people with higher RR scores was also reported in a previous study (3). High RR may promote habituation to affective sensory inputs since higher RR likely lowers the threshold of pre-attentive responses to affective inputs. This is consistent with the observation regarding higher sensitivity to warning signals in people with lower impulsivity (12, 15). A previous EEG study reported that SG of P50 significantly correlated with the ratio of estimated hippocampal source activities in healthy populations (22). A previous PET study also demonstrated that aversive auditory stimuli activated the prefrontal (dorsolateral, superior, and medial frontal), inferior parietal, and limbic system, including the amygdala, hippocampus, and insula (66). Deactivations of the dorsolateral prefrontal cortex, hippocampus, insula, and thalamus were observed for P50 during SG (25, 26). Unusual activations of hippocampus and thalamus were also observed in patients with schizophrenia (31). Based on these source findings, habituation to aversive noise likely reduced activity in these areas, inducing SG of P50, as observed in people with higher-scoring RR in the present study.

The P200 ratio in the white noise condition negatively correlated with the self-reported fun-seeking. Because fun-seeking scores positively correlated with RR scores (*r* = 0.508, *p* = 0.013), high fun-seeking behaviors may also be associated with the habituation toward affective sensory inputs, and may likely deactivate the cortical areas related to the SG of P50 (e.g., dorsolateral prefrontal areas, hippocampus, and insula). The P50 ratio positively correlates with the P200 ratio (*r* = 0.423, *p* = 0.044), which suggests that SG in early auditory processing affects later attentive auditory processing. Because SG has been observed during later auditory processing stages, particularly in the hippocampus (23), this area may partially modify neural processing of S2 in the auditory cortex associated with P200 elicitation (67).

Significant correlations between the N100 ratio and self-reported impulsivity components were not observed in the present study. Because SG of the N100 ratio in the white noise condition tended to correlate with the P50 ratio (*r* = 0.412, *p* = 0.051) and the P200 ratio (*r* = 0.586, *p* = 0.003), SG of N100 may not be completely dissociated from those of P50 and P200. However, N100 may be primarily related to the activation of the auditory cortex (68), while P50 may reflect a top-down modulated early auditory processing, which is related to activation of not only the auditory cortex, but also the prefrontal cortex (23). Such differences in neural modification by other areas during auditory processing may contribute to the differences in SG between N100 and P50 as well as P200.

The frequency-domain analyses demonstrated that a smaller ERD of the β band for the S2 in the white noise condition was related to lower self-reported MI. Although there was differences in impulsivity subcomponents associated with SG changes between the time-domain and frequency-domain analyses, the results of the β band also suggest that SG for white noise is associated with attentional change by MI. Kamiński et al. (69), for example, reported that greater power in the β band was associated with increased attention, and also correlated with faster reaction time during visual discrimination tasks. Gola et al. (70) showed that β band activities in younger and high-performance older adults were related to correct, attentive performances in a visual cue-target verification task, while attention-deficit older adults did not show increased β band activity. Although neither the S1 nor the S2 of the white noise condition in the present study increased synchronization (ERS) activity in the β band, participants with lower-scoring MI showed smaller ERD (more positive difference scores) of the β bands for S2 compared with S1. This suggests that people with lower MI traits may become less accustomed to, and thereby pay more attention to, repeated white noise. In contrast, the change in β band activity for the pure tone condition did not significantly correlate with impulsivity scores, but the ERD of β band activity was smaller in S2, compared to S1. This suggests that the successive presentation of pure tones gradually increases attentional orientation to stimuli, irrespective of impulsivity traits. To summarize, pure tones and white noise may stimulate cortical attentional networks differently in a temporal manner, yielding different parametric modulations with different MI levels.

The δ band activity during pure tones positively correlated with BAS scores, which assess traits of extraversion, a known aspect of impulsivity (71). Although attentional modulation of δ band activity has not been clearly established in previous studies, lower frequency band activity (e.g., θ band) decreased with an increase in attentive attitudes in healthy people (70). Thus, in contrast to the β band activity, a smaller or more positive ERD of the δ band for S2, compared to S1, is related to higher BAS scores with reduced attention. A pure tone was a less affective warning signal than white noise (36), and therefore, it might easily generate a greater decrease of attention and reduced arousal states in people with higher BAS scores. To summarize the frequency-domain findings, because the β and δ bands demonstrated different changes in desynchronization for S2 in either the pure tone or white noise condition, attentional modulation in sensory inhibition may be a multifaceted phenomenon related to multiple cortical (25, 26) and temporal domains.

Finally, we should refer to general differences in evoked neural activities between a pure tone and white noise. Irrespective of sensory inhibition properties, the N100 and P200 amplitudes for white noise were generally smaller than those for pure tones, as indicated by the main effect of ST in the overall ANOVA. The differences between the two sounds may result from differences in neural coding in the auditory cortex. White noise has a temporally and spectrally uncorrelated structure, while a pure tone possesses a temporally correlated oscillatory structure (72). Such structural differences may yield differences in neural coding; white noise, as opposed to a pure tone, may not clearly show phase-locking neural responses, and may not constantly generate maximum neural responses, while showing an onset burst of neural response to broadband frequencies (73). Such differences in neural coding are likely related to the differences in averaged neural responses of the N100 and P200 between white noise and pure tones. Additional studies will be necessary to elucidate how the differences in neural coding between pure tones and white noise are related to the SG for emotional sounds.

Despite the various findings argued above, there are the limitations in the present study. First, we used self-report scales to assess impulsivity traits. Self-report may be biased and not necessarily correlate with experimental (e.g., a response inhibition task) or neurobiological measures of impulsivity traits, especially in clinical populations (1, 74, 75). Additionally, non-pathological impulsivity may not be easily comparable to pathological impulsivity using self-report scales, as suggested by group differences in correlations between SG and self-reported impulsivity (3). Future studies should use not only self-report but also objective measures of impulsivity traits, in particular when investigating neural correlates of pathological impulsivity in clinical populations. Second, the present sample size was relatively small, and the gender was intermingled while we also tested partial correlations between SG and impulsivity scores, using the covariates of the gender as well as age. To examine possible sex effects on SG (56) changing with impulsivity, future studies should compare equally larger samples of female and male participants with similar socio-demographic profiles.

## Conclusion

To our knowledge, the present study is the first to report that early sensory inhibition to white noise is sensitive to individual differences in self-reported impulsivity in a non-clinical population. White noise sensitivity induced individual variations in sensory inhibition in the time-domain analysis, and higher-scoring impulsivity subcomponents were associated with greater sensory inhibition, likely because higher impulsivity is related to less attentional direction to warning signals. The frequency-domain analyses also suggested attentional modulation of sensory inhibition for white noise, in that a greater attenuation of β band desynchronization was observed for the second white noise in people with a lower-scoring impulsivity subcomponent. Certain clinical populations with pathological impulsivity conversely enhanced early sensori-perceptual neural processing of deviant warning signals, such as an angry voice (76). Our white noise paradigm may also elucidate clear contrasts between non-clinical and clinical populations in early sensory inhibition. Future studies, including clinical populations, will investigate sensory inhibition of white noise to examine the temporal causal relationship between automatic sensory inhibition and subsequent cognitive control to prevent impulsive actions.

## Author Contributions

The present study has been conceived and designed by TS, TN, KA, KN, HT, and TO. The experiments were performed by TS, KN, KA, and HT. Data were analyzed by TS, KN, and HT. Data have been interpreted by TS, TN, KA, and TO. The manuscript was written, revised, and approved by TS, TN, KA, and TO.

## Conflict of Interest Statement

The authors declare that the research was conducted in the absence of any commercial or financial relationships that could be construed as a potential conflict of interest.

## Funding

This study was supported by an Intramural Research Grant (24-3) for Neurological and Psychiatric Disorders of NCNP.
